# Genome-Wide Identification of the *Remorin* Gene Family in Poplar and Their Responses to Abiotic Stresses

**DOI:** 10.3390/life14101239

**Published:** 2024-09-27

**Authors:** Zihui Li, Hang Wang, Chuanqi Li, Huimin Liu, Jie Luo

**Affiliations:** 1 College of Horticulture and Forestry Science, Hubei Engineering Technology Research Center for Forestry Information, Huazhong Agricultural University, Wuhan 430070, China; lizihui0428@163.com (Z.L.); wangghangg@163.com (H.W.); lichuanqi@binn.cas.cn (C.L.); 2 Research Institute of Non-Timber Forestry, Chinese Academy of Forestry, Zhengzhou 450003, China

**Keywords:** bioinformation, gene expression, *Populus trichocarpa*, *Remorin* gene family

## Abstract

The *Remorin* (*REM*) gene family is a plant-specific, oligomeric, filamentous family protein located on the cell membrane, which is important for plant growth and stress responses. In this study, a total of 22 *PtREMs* were identified in the genome of *Populus trichocarpa*. Subcellular localization analysis showed that they were predictively distributed in the cell membrane and nucleus. Only five PtREMs members contain both Remorin_C- and Remorin_N-conserved domains, and most of them only contain the Remorin_C domain. A total of 20 gene duplication pairs were found, all of which belonged to fragment duplication. Molecular evolutionary analysis showed the *PtREMs* have undergone purified selection. Lots of *cis*-acting elements assigned into categories of plant growth and development, stress response, hormone response and light response were detected in the promoters of *PtREMs*. *PtREMs* showed distinct gene expression patterns in response to diverse stress conditions where the mRNA levels of *PtREM4.1*, *PtREM4.2* and *PtREM6.11* were induced in most cases. A co-expression network centered by *PtREMs* was constructed to uncover the possible functions of *PtREMs* in protein modification, microtube-based movement and hormone signaling. The obtained results shed new light on understanding the roles of *PtREMs* in coping with environmental stresses in poplar species.

## 1. Introduction

Remorin (REM) is a plant-specific protein associated with the plasma membrane (PM) microdomain (lipid raft) with strong hydrophilicity, which responds to oligogalacturonic acid signals [1,2,3]. *REMs* encode an oligomeric, filamentous protein family formed by coiled coils and are expressed in the leaves, branches, stems and roots of plants with strong division ability [4]. Recent studies have shown that *REMs* have a variety of biological functions in plant growth and development [5,6,7] and are also involved in hormone signaling pathways [8], disease resistance [9], stress response [10,11] and plant immunity [12,13]. Therefore, *REMs* could serve as molecular targets for improving plants’ adaptation to various abiotic and biotic stresses.

REM contains a highly conserved C-terminal domain and a significantly variable N-terminal domain [1]. The C-terminal domain of REM has a highly conserved coiled-coil structure with hydrophobicity [3]. The coiled-coil structure is a hypothetical membrane-anchored motif involved in the regulation of the spatial structure and oligomerization of the REM [14,15,16,17]. In addition, the C-terminal of REM also has a short sequence of REM-CA (REMORIN C-terminal Anchor), which mediates the binding of REM to PM and participates in protein-protein interactions [18,19]. REM also has a conserved region that is rich in proline [3]. Most REMs are rich in cysteine, which is a potential isoprene reaction site and mediates the interaction between REM and PM [1,20]. The N-terminal intrinsically disordered domain (IDD) is involved in protein aggregation and lipid interaction [19,21]. The sequence variations of REM proteins in the Remorin-N domain lead to diversity in their structure and biological functions [4].

In rice, the *REM* gene *Grain setting defect1* (*GSD1*) inhibits the transport of carbohydrates from the photosynthetic site to the phloem and affects seed setting by regulating intercellular filament conduction [22,23]. *REM1.2* can impair the movement of carrot mosaic virus cells by interacting with PM-associated Ca^2+^ binding protein 1 (PCaP1) [24]. Palmitoylation of NbREM1.5 can regulate plasmodesmata permeability and limit the movement of tobacco mosaic virus (TMV) [25,26]. CaREM1.4 can interact with CaRIN4 (RPM1-interacting protein 4) to promote cell death, thereby regulating the tolerance to *Ralstonia solanacearum* [27]. Heterologous expression of *Solanum lycopersicum SlREM1* gene in *Nicotiana benthamiana* increased ROS accumulation and triggered programmed cell death in transgenic plants [28,29]. The symbiosis-specific *SYMREM1* also plays an important role in stabilizing the topological structure of membranes [30]. However, information about REM in woody plants remains scarce.

Poplar, a deciduous tree belonging to the Salicaceae with high economic and ecological values, is widely used to produce wood, reduce soil erosion, purify air and water quality, and protect the environment and ecological ecosystems [31,32,33,34,35,36]. With the completion of the whole genomes of poplar species [37,38] and easy genetic transformation [39], poplar species have become the model plant for molecular breeding for woody plants [40,41]. Further, the functions of some *REMs* in poplar have been reported in recent years [42,43]. The systematic analysis of the poplar *REM* gene family and their roles in regulating poplar growth and development, as well as stress response, remain to be explored. In this study, the *PtREM* gene family was identified by bioinformatics methods using the newly released *Populus trichocarpa* genome (v4.1). Comprehensive bioinformatic analyses have been carried out to explore the potential roles of *PtREMs* in poplar growth and environmental adaptation. The results obtained from this study will provide a basis for improving tree growth and stress resistance by genetically manipulating *PtREMs*.

## 2. Materials and Methods

### 2.1. Identification of the PtREM Gene in P. trichocarpa

The identification of PtREMs in *P. trichocarpa* was conducted according to the methods of the previous reports [44,45]. First, the *Arabidopsis thaliana* database TAIR (https://www.arabidopsis.org/, accessed on 25 August 2024) was used to obtain the protein sequences of the *AtREM* gene family. The whole genome of *P. trichocarpa* (v4.1) and annotation files were taken from the Phytozome database (v13, https://phytozome-next.jgi.doe.gov/, accessed on 25 August 2024). A local BLASTP was conducted using the amino acid sequences of *AtREM* as queries against the *P. trichocarpa* protein database with an E-value less than 10^−5^. The PFAM database [46] was used to evaluate the conserved protein domains of AtREM, and the hidden Markov model (HMM) files for Remorin_C (PF03763) and Remorin_N (PF03766) were retrieved. Using HMMER3.0 (http://www.hmmer.org/, accessed on 25 August 2024), the HMM was built and then examined against the *P. trichocarpa* protein database. All relevant sequence data that had an E-value of less than 10^−5^ were retained. After the findings from HMMER and BLASTP were combined, candidates for REM protein members were submitted to the HMMER database and the NCBI database (CDD) to verify conserved protein domains [47,48].

### 2.2. Bioinformatics Analysis

Using the PhytoMine tool on the Phytozome website (https://jgi.doe.gov, accessed on 26 August 2024), amino acid sequence analyses of *PtREMs* were carried out [49]. The gene models of 22 *PtREMs* were entered to determine the loci, chromosome position and amino acid number of the *PtREM* gene family in *P. trichocarpa*. The physicochemical properties of PtREMs were calculated using the ProtParam (https://web.expasy.org/protparam/, accessed on 26 August 2024) based on their amino acid sequences. The subcellular location of PtREMs was predicted using Plant mPLoc (version 2.0) [50] and the gene and protein structures were visualized using Tbtools (v 2.099) [51,52].

### 2.3. Phylogenetic and Sequence Analysis

A total of 38 protein sequences from *P. trichocarpa* and *A. thaliana* were acquired to construct the phylogenetic tree [1]. The ClustalW tool was used for sequence alignment and the maximum likelihood method was used to build the phylogenetic tree in MEGA software (version 11.0.13) [53]. The phylogenetic tree was visualized on the iTOL website (https://itol.embl.de/, 26 August 2024) [54].

### 2.4. Analysis of Cis-Acting Element

The 2000 bp sequences upstream of the start codon of *PtREM* family members were retrieved from the Phytozome website (https://jgi.doe.gov, accessed on 1 September 2024) [49] and submitted to the PlantCARE online website (https://bioinformatics.psb.ugent.be/webtools/plantcare/html/, accessed on 1 September 2024) for *cis*-acting element analysis and prediction [55].

### 2.5. Gene Duplication Events Analysis

The MCScanX software [56] was employed to detect gene duplication pairs of *PtREM* family members in *P. trichocarpa*. The TBtools software (v 2.099) [51,52] was used to compute the Ka/Ks values of gene replication pairs. Using the Advanced Circos tool in TBtools (v 2.099) [51,52], the collinearity maps of the *PtREM* gene family members in *P. trichocarpa* and among several plant species were produced.

### 2.6. Analysis of Gene Expression

The gene expression levels of *PtREM* gene family members in different treatments and tissues were downloaded from the Phytozome database [49].

The transcriptomes of *P. trichocarpa* under different stress treatments were downloaded from the SRA database under accession of PRJEB19784 with treatment as described previously [57]. Analyses of these stress transcriptomes were provided in the previous study [58]. The heatmaps of gene expression were performed with the pheatmap package [59] in R Studio (version 2023.12.1 Build 402).

### 2.7. Co-Expression Network Analysis

The top 50 most correlated genes of each *PtREM* in *P. trichocarpa* GeneAtlas (V2) were retrieved from the Phytozome website and considered as co-expression genes to construct the transcriptional co-expression network of *PtREM* family members in *P. trichocarpa*. The co-expression network was then visualized using Cytoscape software (version 3.8.2) [60]. Using the clusterProfiler package (version 4.12.0) [61], analyses of Gene Ontology (GO) and Kyoto Encyclopedia of Genes and Genomes (KEGG) were carried out in R and displayed as bubble diagrams, as suggested in the previous publications [62,63].

## 3. Results

### 3.1. Identification and Physicochemical Properties of PtREM Members

A total of 22 PtREM proteins were identified in the *P. trichocarpa* (V4.1) genome using the BLASTP and HMMER techniques (Table 1). Variations in the number of amino acids, molecular weight (MW), isoelectric point, and other features were examined (Table 1). The 22 PtREMs had protein lengths ranging from 124 aa to 606 aa; PtREM5.1 and PtREM3.1, respectively, had the longest and shortest encoding proteins (Table 1). The MW of the PtREM proteins ranged from 14.47 to 66.83 kDa, and 87% of them had isoelectric points larger than 7, indicating that the majority of PtREM proteins were alkaline proteins (Table 1). Furthermore, subcellular localization analyses of PtREM in *P. trichocarpa* revealed that six PtREM proteins were predictively found in the cell membrane, three in the nucleus, and thirteen in both the nucleus and the cell membrane (Table 1).

The 22 *PtREMs* were evenly distributed on 11 of the 19 chromosomes in the *P. trichocarpa* genome, with 1–3 genes on each chromosome, according to the chromosome distributions of *PtREMs* (Table 1). For example, *PtREM2.1*, *PtREM6.3*, and *PtREM6.2* were located on Chr01, *PtREM6.1*, *PtREM6.6*, and *PtREM6.7* were located on Chr08, *PtREM6.5*, *PtREM6.8* and *PtREM6.10* were distributed on Chr10, and *PtREM1.3*, *PtREM5.2*, and *PtREM6.11* were distributed on Chr14 (Table 1).

### 3.2. Structural and Phylogenetic Analysis of PtREMs

**Figure 1:**
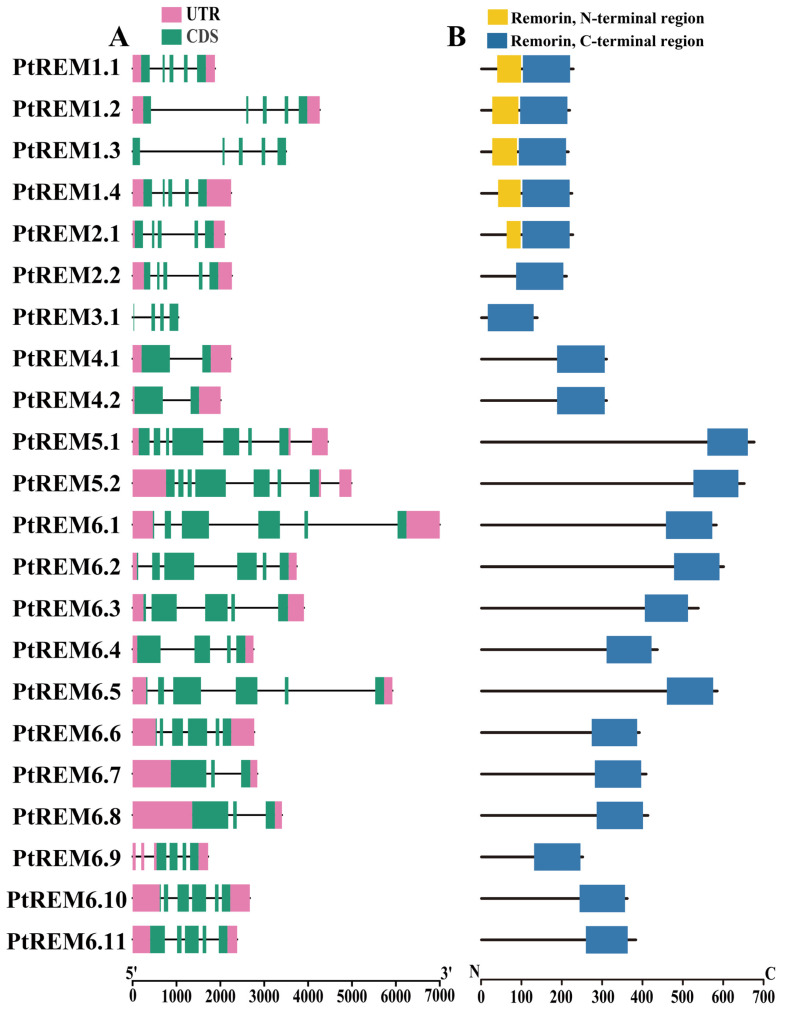
The gene (**A**) and protein (**B**) structures of PtREMs in *P. trichocarpa*. (**A**) Exon–intron structure of *PtREMs* in *P. trichocarpa*. Purple represents the UTR; green denotes CDS; and the black line represents introns; (**B**) analysis of the conserved domains of PtREM proteins. Yellow indicates the Remorin_N domain and blue indicates the Remorin_C domain.

The gene architectures of the *PtREM* gene family varied greatly (Figure 1). Except for *PtREM1.3* and *PtREM3.1*, the majority of *PtREMs* had 5′ and 3′ untranslated regions (UTR) (Figure 1A). While *PtREM4.1* and *PtREM4.2* only had one intron and two exons, *PtREM3.1* only had 124 amino acids and the shortest coding sequences (CDS) (Figure 1A). *PtREM5.1* and *PtREM5.2* had the highest number of exons, whereas *PtREM1.2*, *PtREM1.3*, *PtREM6.1*, and *PtREM6.5* had the longest introns (Figure 1A). The diverse gene structures of *PtREMs* implied that this gene family has experienced functional differentiation events during its evolutionary history. Regarding protein structures, the remaining PtREMs exclusively had Remorin-C regions; only PtREM1.1–1.4 and PtREM2.1 possessed both Remorin-C and Remorin-N domains (Figure 1). Consequently, the information implied that the Remorin-C regions of PtREM played important roles in their biological functions in poplar.

**Figure 2:**
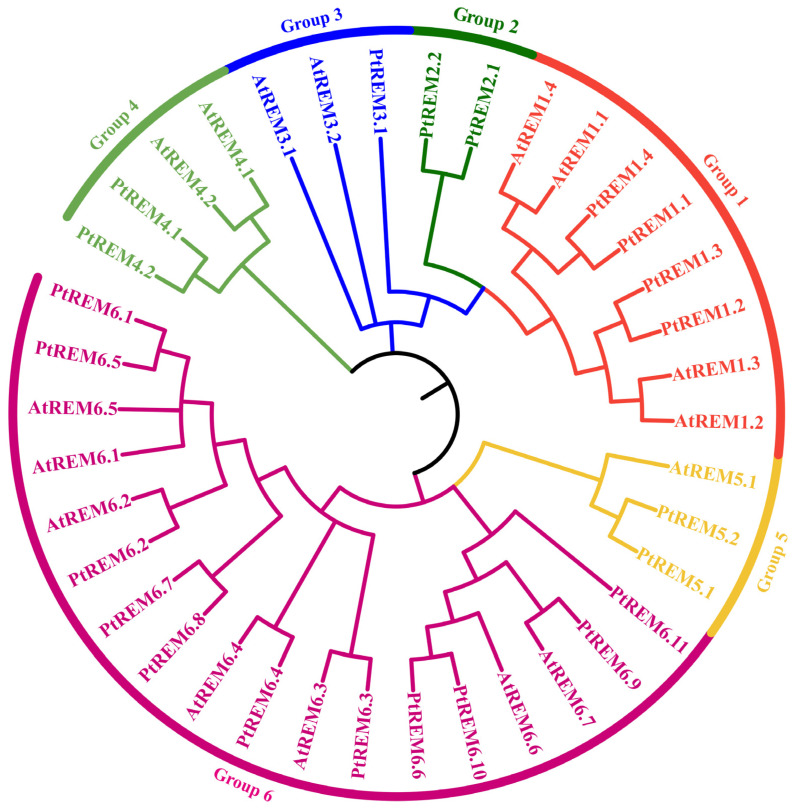
Phylogenetic analysis of REMs in *Arabidopsis* and *P*. *trichocarpa*. Different colored lines represent different groups.

A maximum likelihood phylogenetic tree consisting of 38 REM proteins was constructed, with 22 and 16 REMs in *P. trichocarpa* and *A. thaliana*, respectively (Figure 2). In line with earlier research, the *REM* genes were categorized into six groups: *PtREM1.1–PtREM1.4* were in group 1, *PtREM2.1–PtREM2.2* were in group 2, *PtREM3.1* were in group 3, *PtREM4.1* and *PtREM4.2* were assigned to group 4, *PtREM5.1–PtREM5.2* were in group 5, and a total of eleven *PtREM6s* (*PtREM6.1–PtREM6.11*) were grouped in the same branch in group 6 (Figure 2). Groups 5 and 6 were tightly allocated together among the six groups, indicating that these two groupings had close phylogenetic ties (Figure 2).

### 3.3. Analysis of Gene Duplication Events

**Figure 3:**
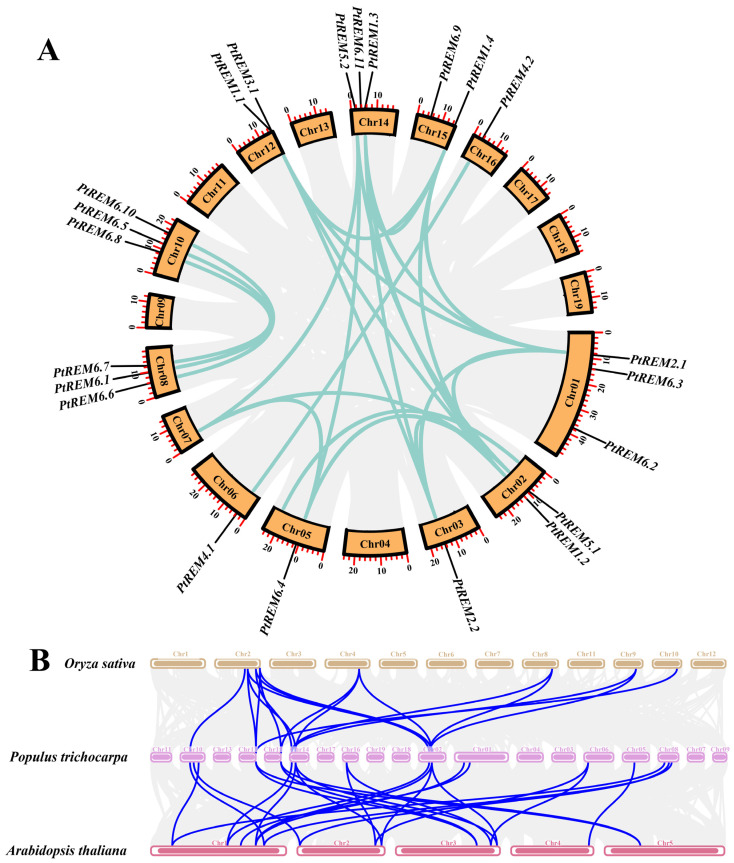
Analysis of collinearity relationships of *REM* in *P. trichocarpa* (**A**) and among different plant species (**B**). (**A**) Collinearity analysis of *PtREMs* in *P. trichocarpa*. Repeated *PtREM* gene pairs were ligated with bluish-green lines. (**B**) Synteny analysis of *REM* genes in *P. trichocarpa*, *A. thaliana*, and *O. sativa*. The gray lines in the background represent the collinearity in the genomes of *P. trichocarpa* and other plant species, and the blue lines highlight the collinearity of the *REM* genes.

Gene duplication events are classified into two categories based on the physical location of replication pairs. Tandem duplications are defined as gene duplications that occur between adjacent positions on the same chromosome while segmental duplications are defined as gene duplications that occur at physical positions greater than 5 Mb or even on different chromosomes [64]. Chromosome mapping has revealed that 22 *PtREM* family members of *P. trichocarpa* are spread across 11 chromosomes, all of which belonged to fragment replication. The majority of *PtREMs* were found on Chr01, Chr10, and Chr14 (Table 1 and Figure 3).

The 22 *PtREMs* in the *P. trichocarpa* genome contained a total of 20 gene duplication pairs that belonged to segmental duplications according to the results of paralogous gene analysis (Figure 3A). Between Chr10 and Chr8, a total of three pairs of gene duplications were found: *PtREM6.10*/*PtREM6.6*, *PtREM6.5*/*PtREM6.1*, and *PtREM6.8*/*PtREM6.7*, respectively (Figure 3A). Based on the calculation of 20 gene duplication pairs of *PtREMs*, the Ka/Ks values ranged from 0.11 to 0.76 (Supplementary Table S1), suggesting that most *PtREMs* had experienced substantial purification selection during their evolutionary history. Furthermore, between *P. trichocarpa* and *Arabidopsis*, 24 collinearity gene pairs were discovered, and between *Oryza sativa* and *P. trichocarpa*, 16 *REM* collinearity gene pairs were discovered (Figure 3B).

### 3.4. Tissue Specific Expression Analysis of PtREM Genes in P. trichocarpa

**Figure 4:**
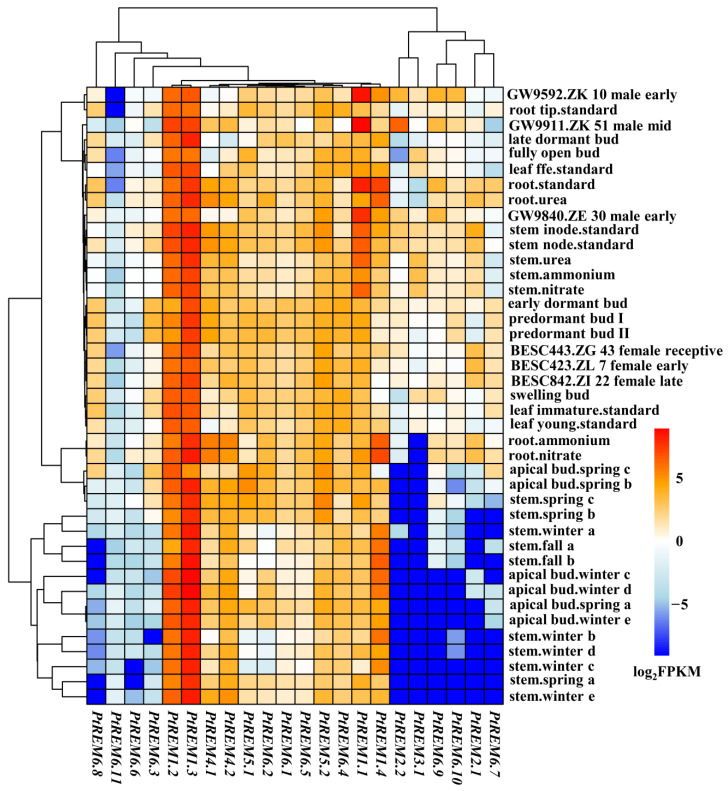
The expression of *PtREMs* in different tissues and different treatments. Orange bars indicate upregulation and blue bars indicate downregulation.

The expression levels of *PtREMs* in different tissues across growth seasons were retrieved from the Phytozome database (Figure 4). Tissue-specific expression profiles were detected for most *PtREMs* across the growth season (Figure 4). For instance, seven *PtREM6s* (i.e., *PtREM6.3* and *PtREM6.6–PtREM6.11*), as well as *PtREM2.1–PtREM3.1*, were seldom expressed in stems and apical buds across the growth season while moderately expressed in the remaining tissues, except for *PtREM6.6* and *PtREM6.11* (Figure 4). *PtREM1.2* and *PtREM1.3* were highly expressed among all the detected different tissues (Figure 4); the remaining *PtREMs* showed no significant tissue specificity (Figure 4). This tissue-specific expression pattern provides a preliminary clue for the function of *PtREMs* in plants and lays the foundation for further exploration of the function of *PtREMs*.

### 3.5. Cis-Acting Element Analysis

**Figure 5:**
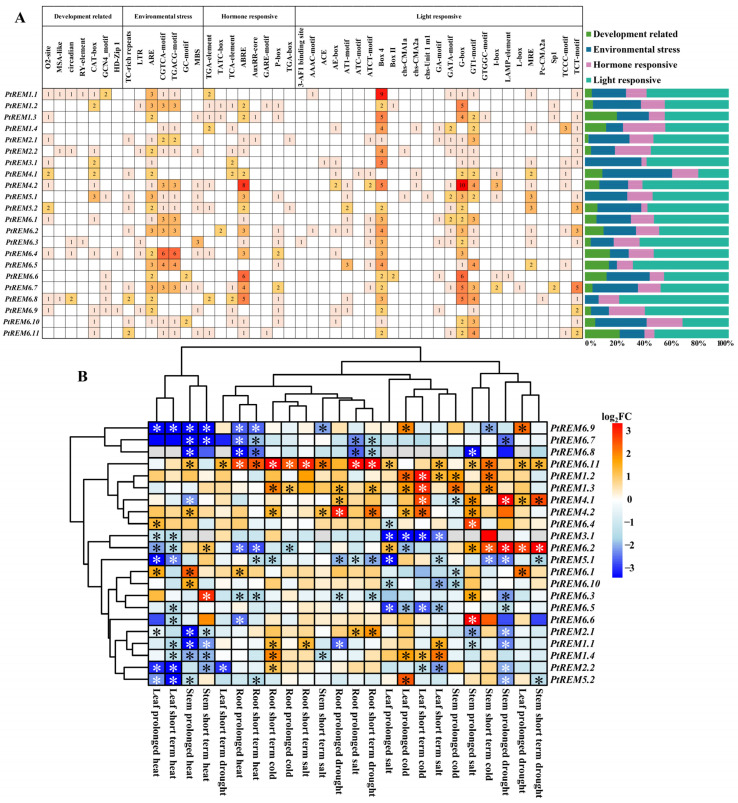
The *cis*-acting element analysis of *PtREMs* in *P. trichocarpa* (**A**) and the expression of *PtREMs* under different stresses (**B**). The number of different promoter elements in the *PtREMs* is represented by different intensity colors and numbers. The different colors in the histogram represent the percentage of *cis*-acting elements in the four functional categories. In the heatmap, orange and blue colors indicate upregulation and downregulation, respectively. The stars in cells indicate significance.

To study the possible regulation of *PtREMs* in *P. trichocarpa*, the *cis*-acting elements of *PtREM* promoters were analyzed (Figure 5A). The results showed that many *cis*-acting elements related to plant growth and development, stress, hormone response and light response were found in *PtREM* promoters (Figure 5A). For plant growth and development elements, most *PtREMs* contain O_2_ sites involved in the regulation of zein metabolism and in the *cis*-acting element CAT-box upstream of eukaryotic structural genes (Figure 5A). As for the stress response elements, the anaerobic induction essential element ARE and the CGTCA-motif and TGACG-motif involved in the MeJA response were ubiquitous in the promoters of *PtREMs* (Figure 5A), which implies that *PtREM* may be involved in stress response. As for hormone-responsive *cis*-elements, most *PtREMs* contain ABRE involved in abscisic acid response and TCA-element involved in salicylic acid response (Figure 5A), indicating that *PtREM* might be regulated by multiple hormone signaling pathways. The promoters of *PtREM* also generally contained Box4, G-Box and GT1-motif light-responsive *cis*-acting elements, implying that *PtREMs* may be regulated by light signaling (Figure 5A). Among the detected *cis*-acting elements, the *cis*-acting elements assigned as light responses occupied ca. 21.21–76.19% of all detected *cis*-acting elements for all the *PtREMs* (Figure 5A) while ca. 4.17–4.76% *cis*-acting elements belonging to development regulation were detected for *PtREM1.3* and *PtREM6.11* (Figure 5A), suggesting potential roles of these two genes in poplar development processes. Additionally, eight *cis*-acting elements of ABRE were detected in the promoter of *PtREM4.2* (Figure 5A). Taken together, the obtained data suggest *PtREMs* play an important role in plant growth and development, stress, hormone responses and light responses.

As considerable *cis*-acting elements related to stress response, the transcriptional expression levels of *PtREMs* responding to diverse stress conditions in different tissues were analyzed using online transcriptome data (Figure 5B). *PtREM6.7–PtREM6.9* showed similar expression profiles to diverse stress conditions, which were inhibited by prolonged and/or short-term heat stress in roots, stems and leaves for most cases (Figure 5B). The duplication gene pairs of *PtREM1.2* and *PtREM1.3* were significantly induced by prolonged and/or short-term cold stress in leaves and stems of *P. trichocarpa* (Figure 5B). In addition, the *PtREM4.1*/*PtREM4.2* gene duplication pair, whose mRNA levels were significantly induced by prolonged salt in stems, short-term cold in leaves, and prolonged drought in roots, showed similar gene expression patterns in response to diverse stress conditions (Figure 5B). Additionally, the mRNA levels of *PtREM6.11* were significantly stimulated by most detected stress conditions, while *PtREM5.1* displayed the opposite responses (Figure 5B). The foliar mRNA levels of *PtREM3.1* and *PtREM6.5* were significantly downregulated to prolonged and/or short-term heat, salt and cold stresses (Figure 5B). Finally, two gene duplication pairs of *PtREM1.1*/*PtREM1.4* and *PtREM2.1*/*PtREM2.2,* together with *PtREM5.2,* were assigned to the same cluster with significantly decreased mRNA levels under short-term drought in stems and under prolonged and/or short-term heat stresses in leaves and stems of *P. trichocarpa* (Figure 5B). Additionally, *PtREM1.1* and *PtREM1.4* were induced by short-term salt in leaves and by short-term cold in roots (Figure 5B).

### 3.6. Co-Expression Network Analysis of PtREMs

**Figure 6:**
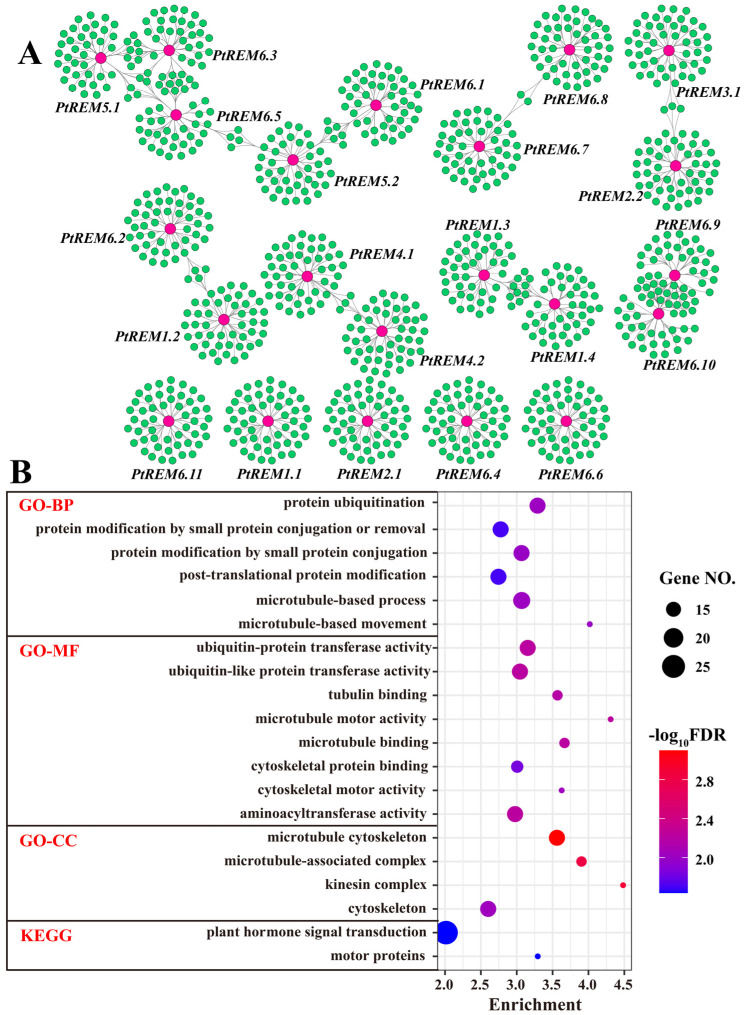
Co-expression network of *PtREMs* in *P. trichocarpa* (**A**), as well as the GO and KEGG enrichment analyses of genes in the co-expression network (**B**). In the co-expression network, the red and green nodes represent *PtREMs* and their co-expressed genes, respectively. The edges of the network indicate the co-expression relationships between *PtREMs* and their co-expressed genes.

To uncover the possible biological functions played by *PtREMs*, a transcriptional co-expression network has been constructed by a total of 1035 genes with 1100 interactions (Figure 6A). Five *PtREMs* (i.e., *PtREM2.1*, *PtREM5.2*, *PtREM6.1*, *PtREM6.3*, and *PtREM6.5*) shared certain common co-expression genes to form the biggest module in the co-expression network (Figure 6A). Additionally, gene duplication pairs of *PtREM4.1*/*PtREM4.2* and *PtREM6.7*/*PtREM6.8* also had some common co-expressed genes (Figure 6A). Several genes from the same subgroup also shared lots of common co-expressed genes, such as *PtREM2.2* and *PtREM3.1*, *PtREM1.3* and *PtREM1.4*, as well as *PtREM6.9* and *PtREM6.10* (Figure 6A). None of the shared, co-expressed genes with other *PtREMs* were detected for *PtREM1.1*, *PtREM2.1*, *PtREM6.4*, *PtREM6.6* and *PtREM6.11* (Figure 6A). The GO and KEGG enrichment analysis of genes in the *PtREMs* co-expressed network implied roles of *PtREMs* in protein ubiquitination and modification, plant hormone signal transduction, ubiquitin-protein transferase activity, and microtubule-based movement (Figure 6B).

## 4. Discussion

REM is a plant-specific protein located on the PM, which plays an important role in plant growth and stress response [4]. In recent years, the *REM* gene family has been identified in many species, such as *A. thaliana*, *O. sativa*, *Brassica napus*, *Setaria italica* and *Saccharum* [1,10,65,66]. A total of 22 *PtREMs* were identified in poplar; 19 of them were predictively cellularly distributed on the cell membrane, which implied that PtREMs might affect the structure of the PM to a certain extent [20]. Two *PtREM2* members were found in the genome of *P. trichocarpa*, which was consistent with the earlier findings that the *REM2* members were only detected in legumes and poplar species [1,10].

Only five of the PtREMs contain both Remorin-C and Remorin-N domains while the rest ones only contain the Remorin-C conserved domain, suggesting that the Remorin-C conserved domain is essential for their functions. Consistent with existing knowledge, the C-terminal structure of REM proteins is stable while the N-terminal structure changes greatly [1]. The C-terminal region of the REM, as a signature region of the *REM* gene family, has a highly conserved coiled-coil structure and hydrophobicity [3] and plays an important role in both the binding of the REM to the PM and the interaction between the proteins [18,19]. Studies have shown that the C-terminal region of AtREM1.3 is essential for stable protein oligomerization, and its N-terminal region promotes the interaction with importin α isoforms (AtIMPα) [67]. Some PtREMs contained only one Remorin-C domain with large variations. Moreover, the differences in N-terminal structures may endow diverse biological functions for plants [19]. Although the members of PtREMs from Group 1 contained both Remorin-N and Remorin-C domains, they showed distinct transcriptional responses to diverse stress conditions, implying that functional differentiation among *PtREM1* has occurred. Interestingly, only one (PtREM2.1) out of two PtREM2s contained the Remorin-N domain. Gene duplication analysis detected two duplication events for PtREM2.1 with PtREM1 members, suggesting that PtREM2.1 may originate from PtREM1.

Tissue-specific analysis of *PtREMs* showed that all the members of *PtREM1*, *PtREM4* and *PtREM5* were constitutively high or moderately expressed in different tissues, suggesting the essential roles played by these genes in normal plant growth and development. In line with this, *PdREM* from *P. deltoides* regulates vascular growth and wood properties [42]. Additionally, *PtREM3.1* and *PtREM6.7–PtREM6.11* showed low expression in apical buds and stems across the growth seasons. In the fruits of *Mangifera indica* L., the *REM* genes were stimulated by cold stress in a brassinolide-dependent manner [68], highlighting the importance of PM proteins in coping with cold stress for plants. In current studies, *PtREM1.1–PtREM1.4*, *PtREM2.2*, *PtREM4.1*, *PtREM4.2*, *PtREM6.2*, *PtREM6.9* and *PtREM6.11* were stimulated in at least one tissue by cold stress, suggesting that these genes played important roles in stabilizing PM structures to allow them to cope with cold stress. The expression levels of *AtREM4.1* and *AtREM4.2* significantly increased after stress treatment and plant hormone treatment [1]. Accordingly, the expression levels of *PtREM4.1* and *PtREM4.2* in the same group also increased after diverse stress treatments, and the *PtREM4.2* promoter contains many stress and hormone response *cis*-acting elements. Therefore, the roles of *PtREM4.1* and *PtREM4.2* in improving the stress resistance in *P. trichocarpa* could be expected, though further studies are needed to verify this.

Upon different stress treatments, the gene expression patterns of the genes from the same phylogenetic branch were highly similar, especially for gene duplication pairs. For example, *PtREM1.2* and *PtREM1.3* were greatly affected by cold stress and the expression was significantly upregulated after cold stress treatment. *PtREM4.1* and *PtREM4.2* were upregulated after drought and cold stress treatment. The expression levels of *PtREM6.4* and *PtREM6.6* in stems were increased after long-term salt treatment. Gene co-expression network analysis also uncovered that gene duplication pairs of *PtREM4.1*/*PtREM4.2* and *PtREM6.7*/*PtREM6.8* shared certain common co-expressed genes. These results suggest that some gene duplication pairs of *PtREMs* from the same group may have similar functions in response to environmental stresses. However, the *PtREM2.1* showed distinct gene expression profiles with its duplicate gene pairs (i.e., *PtREM1.2* and *PtREM1.3*), suggesting that there are different roles played by *PtREM2.1* and its duplicate pairs in the *PtREM1* group in response to environmental stresses.

Salt stress is one of the most serious abiotic stresses, which has serious negative effects on plant growth and ecosystem productivity worldwide [69]. In foxtail millet (*S. italica*), the expression levels of *SiREM6* were significantly induced by high salt stress, and overexpression of *SiREM6* in Arabidopsis improved the plants’ tolerance to salt stress [70]. Similarly, overexpression of *P. euphratica REM6.5* in Arabidopsis activates the activities of PM H^+^-ATPase to improve the tolerance to salt stress in transgenic plants [71]. Further, overexpression of mulberry (*Morus indica*) *MiREM* in Arabidopsis improved the growth performance under drought stress conditions [11]. Accordingly, the mRNA levels of *PtREM4.1*, *PtREM4.2* and *PtREM6.11* were significantly stimulated by the salt, drought and cold stresses together with several *cis*-acting elements related to stress response on the promoters of these genes, suggesting their potential roles in coping with salt stress. Therefore, *PtREM4.1*, *PtREM4.2* and *PtREM6.11* could serve as molecular targets for improving plants’ resistance to environmental stresses.

## 5. Conclusions

A total of 22 *PtREM* family members were identified in the genome of *P. trichocarpa*, which were evenly distributed on 11 chromosomes. All PtREM proteins contained the conserved Remorin-C domain and only PtREM1.1-PtREM1.4 and PtREM2.1 contained the Remorin-N domain. A total of 20 pairs of gene duplication events were identified in the *PtREM* gene family, all of which belonged to fragment duplication. All the Ka/Ks values of duplicated gene pairs were less than 1, suggesting purification selection has occurred during evolution. Considerable *cis*-acting elements were detected, which could be assigned to plant development, environmental stress, hormone responses and light responses. The transcriptional analysis showed distinct gene expression profiles of the *PtREM* gene family in response to diverse environmental stresses in different tissues. Several duplicated gene pairs of *PtREMs* showed similar gene expression patterns to environmental stresses, implying the functional redundancy of these duplicated gene pairs for environmental adaptation. A co-expression network centered by *PtREMs* was constructed to uncover the possible roles in protein ubiquitination, microtube-based processes and plant hormone signal transduction. Taken together, the obtained results lay a foundation for understanding the essential roles played by *PtREMs* in poplar growth and environmental adaptation, as well as the molecular targets of genetic improvements for abiotic stresses.

## Figures and Tables

**Table 1:** Physicochemical properties and subcellular localizations of the *PtREM* gene family.

Gene Name	Gene Model	Protein (aa)	MW (kDa)	Isoelectric Point	Subcellular Localization	Chromosome Position
*PtREM1.1*	Potri.012G140800	204	22.71	6.00	Cell membrane	Chr12:15283828-15285704
*PtREM1.2*	Potri.002G157700	196	21.31	9.13	Cell membrane	Chr02:12028474-12032739
*PtREM1.3*	Potri.014G081300	193	21.49	6.70	Cell membrane	Chr14:5248038-5251534
*PtREM1.4*	Potri.015G143600	201	22.23	5.09	Cell membrane	Chr15:14900244-14902489
*PtREM2.1*	Potri.001G107000	203	22.71	8.94	Cell membrane	Chr01:8599505-8601605
*PtREM2.2*	Potri.003G124400	189	21.05	7.69	Cell membrane	Chr03:14441701-14443964
*PtREM3.1*	Potri.012G140900	124	14.47	9.15	Cell membrane, Nucleus	Chr12:15289364-15290406
*PtREM4.1*	Potri.006G053200	278	30.55	8.78	Cell membrane, Nucleus	Chr06:3708285-3710532
*PtREM4.2*	Potri.016G054400	278	30.86	6.74	Cell membrane, Nucleus	Chr16:3577080-3579086
*PtREM5.1*	Potri.002G125200	606	66.83	9.64	Cell membrane, Nucleus	Chr02:9533038-9537486
*PtREM5.2*	Potri.014G027900	584	64.13	9.94	Cell membrane, Nucleus	Chr14:1748284-1753282
*PtREM6.1*	Potri.008G144300	522	57.73	9.20	Nucleus	Chr08:9793769-9800768
*PtREM6.2*	Potri.001G358600	538	60.56	8.24	Nucleus	Chr01:37441084-37444823
*PtREM6.3*	Potri.001G163000	482	53.83	8.20	Cell membrane, Nucleus	Chr01:13838091-13841996
*PtREM6.4*	Potri.005G138500	391	42.86	9.66	Cell membrane, Nucleus	Chr05:10893174-10895929
*PtREM6.5*	Potri.010G098000	524	58.07	9.20	Nucleus	Chr10:12130727-12136648
*PtREM6.6*	Potri.008G093300	351	39.07	9.83	Cell membrane, Nucleus	Chr08:5824430-5827202
*PtREM6.7*	Potri.008G178300	366	40.61	9.03	Cell membrane, Nucleus	Chr08:12321723-12324565
*PtREM6.8*	Potri.010G056800	370	41.01	9.04	Cell membrane, Nucleus	Chr10:8762054-8765457
*PtREM6.9*	Potri.015G049700	225	26.26	10.10	Cell membrane, Nucleus	Chr15:5217249-5218970
*PtREM6.10*	Potri.010G160900	324	36.42	10.00	Cell membrane, Nucleus	Chr10:16663990-16669562
*PtREM6.11*	Potri.014G058900	343	38.57	9.51	Cell membrane, Nucleus	Chr14:3792409-3794792

## Data Availability

Data will be available upon request.

## Supplementary Materials

The following supporting information can be downloaded at: https://www.mdpi.com/article/10.3390/life14101239/s1, Table S1. Molecular evolutionary analysis of the *PtREM* genes.
