# Supplementary material for: Genome-Wide Identification of the Remorin Gene Family in Poplar and Their Responses to Abiotic Stresses
Source: Life (Basel). 2024 Sep 27;14(10):1239. doi: 10.3390/life14101239 (PMC11509593; doi:10.3390/life14101239)
Supplement: Supplementary file 1 [file life-14-01239-s001.zip › life-3223426-supplementary.pdf]

Table S1. Molecular evolutionary analysis of the *PtREM* genes.

**Gene pairs**	**Ka**	**Ks**	**Ka/Ks**
PtREM1.1/PtREM1.4	0.0773	0.2064	0.3745
PtREM1.2/PtREM1.3	0.0634	0.3509	0.1807
PtREM1.2/PtREM1.4	0.2131	1.5555	0.1370
PtREM1.2/PtREM2.2	0.3541	1.6606	0.2132
PtREM1.2/PtREM1.1	0.2308	1.4618	0.1579
PtREM1.3/PtREM1.4	0.2233	1.9141	0.1167
PtREM2.1/PtREM1.2	0.3570	1.5623	0.2285
PtREM2.1/PtREM2.2	0.1364	0.1796	0.7596
PtREM2.1/PtREM1.1	0.2528	1.6437	0.1538
PtREM2.1/PtREM1.3	0.3214	2.6033	0.1235
PtREM2.1/PtREM1.4	0.3118	1.7604	0.1771
PtREM2.2/PtREM1.1	0.2746	1.4521	0.1891
PtREM2.2/PtREM1.3	0.2897	2.4752	0.1171
PtREM4.1/PtREM4.2	0.0570	0.3078	0.1852
PtREM5.1/PtREM6.4	0.3703	1.6203	0.2285
PtREM5.1/PtREM5.2	0.0595	0.2527	0.2353
PtREM6.1/PtREM6.5	0.0616	0.2359	0.2610
PtREM6.4/PtREM5.2	0.3616	1.6688	0.2167
PtREM6.6/PtREM6.10	0.1760	0.3435	0.5124
PtREM6.7/PtREM6.8	0.0595	0.2638	0.2254
